# ^129^Xe chemical shift in human blood and pulmonary blood oxygenation measurement in humans using hyperpolarized ^129^Xe NMR

**DOI:** 10.1002/mrm.26225

**Published:** 2016-04-08

**Authors:** Graham Norquay, General Leung, Neil J. Stewart, Jan Wolber, Jim M. Wild

**Affiliations:** ^1^ Unit of Academic Radiology, Department of Cardiovascular ScienceUniversity of SheffieldSheffieldSouth YorkshireUnited Kingdom; ^2^ GE HealthcareAmershamBuckinghamshireUnited Kingdom

**Keywords:** hyperpolarized gases, ^129^Xe spectroscopy, chemical shift, in vivo lung, blood NMR

## Abstract

**Purpose:**

To evaluate the dependency of the ^129^Xe‐red blood cell (RBC) chemical shift on blood oxygenation, and to use this relation for noninvasive measurement of pulmonary blood oxygenation in vivo with hyperpolarized ^129^Xe NMR.

**Methods:**

Hyperpolarized ^129^Xe was equilibrated with blood samples of varying oxygenation in vitro, and NMR was performed at 1.5 T and 3 T. Dynamic in vivo NMR during breath hold apnea was performed at 3 T on two healthy volunteers following inhalation of hyperpolarized ^129^Xe.

**Results:**

The ^129^Xe chemical shift in RBCs was found to increase nonlinearly with blood oxygenation at 1.5 T and 3 T. During breath hold apnea, the ^129^Xe chemical shift in RBCs exhibited a periodic time modulation and showed a net decrease in chemical shift of ∼1 ppm over a 35 s breath hold, corresponding to a decrease of 7–10 % in RBC oxygenation. The ^129^Xe‐RBC signal amplitude showed a modulation with the same frequency as the ^129^Xe‐RBC chemical shift.

**Conclusion:**

The feasibility of using the ^129^Xe‐RBC chemical shift to measure pulmonary blood oxygenation in vivo has been demonstrated. Correlation between ^129^Xe‐RBC signal and ^129^Xe‐RBC chemical shift modulations in the lung warrants further investigation, with the aim to better quantify temporal blood oxygenation changes in the cardiopulmonary vascular circuit. Magn Reson Med 77:1399–1408, 2017. © 2016 The Authors Magnetic Resonance in Medicine published by Wiley Periodicals, Inc. on behalf of International Society for Magnetic Resonance in Medicine. This is an open access article under the terms of the Creative Commons Attribution License, which permits use, distribution and reproduction in any medium, provided the original work is properly cited.

## INTRODUCTION

Knowledge of tissue oxygenation can provide valuable insight into the pathophysiology of a spectrum of diseases. For example, in the discrimination of the penumbra following stroke 1 or identification of ischemia following myocardial infarction 2. Furthermore, hypoxia limits the efficacy of radiotherapy in the treatment of tumors 3. In lung diseases such as asthma and chronic obstructive pulmonary disease, hypoxia can influence the lifetime and the functionality of neutrophils that are associated with inflammation in the lungs 4. Surface blood oxygenation can be measured with an infrared finger probe, but this approach is depth‐limited. The accepted gold standard method of determining deep tissue oxygenation is with polarographic electrodes, as pioneered in the late 1950s 5. This invasive method, however, samples only a small amount of tissue and is able to provide assessment of oxygenation for only limited tissue volumes. Owing to this limitation, oxygenation is usually estimated using surrogate techniques, such as the monitoring of mixed venous oxygenation, heart rate, blood pressure, and oxygen saturation at the jugular bulb. However, these estimates can prove inaccurate as distal tissue oxygenation is not necessarily well represented by the more proximal measurements, and vice versa 6.

MR perfusion imaging can be used to measure blood delivery to a tissue of interest, however, oxygen supply and demand can be independent of one another, e.g., revascularization of fibrotic tissue provides blood flow out of proportion to metabolic demand. Likewise, tissues with low levels of perfusion have an adequate oxygen supply while at rest, but an inadequate oxygen supply during periods of increased metabolic demand. Tissue perfusion alone, therefore, does not completely describe the underlying physiology of gas exchange 7 and thus direct, noninvasive measurement of blood oxygenation may be of interest in many clinical settings, as well as being of interest from a physiological perspective.

Several techniques have been developed to quantify regional oxygenation using MRI. A promising method is the use of blood oxygen level dependent (BOLD) MRI 8, 9, where images that indicate impaired oxygen uptake can be generated, for example by cycling oxygen and carbogen levels 10. However, this technique provides only relative estimates of tissue oxygen partial pressure 11 because there are endogenous variations in magnetization relaxation parameters that can confound these measurements and thus make absolute oxygen concentration detection difficult. Moreover, BOLD signal response is governed by a mixture of $T_{2}^{*}$ dephasing and diffusion due to microscopic susceptibility gradients, and is therefore very much dependent upon the means of measurement, for example the choice of pulse sequence and field strength. Techniques that introduce exogenous tracers for MR oximetry, such as Overhauser‐enhanced MRI of unpaired radicals 12, 13 and ^19^F MRI 14, are promising for measurement of blood oxygenation; however, these tracers have yet to be applied in humans. In the lungs, the $T_{1}$ of inhaled hyperpolarized ^3^He gas in the airspaces 15, 16 has been used to quantify regional alveolar oxygen concentration, but regional ventilation‐perfusion mismatch and impaired gas transfer across the alveolar capillary interstitial barrier means this does not necessarily reflect the capillary blood oxygenation.

Xenon has been in routine clinical use for many years as a tracer of blood perfusion 17, and the physiological effects of xenon gas administration are well known and characterized 18, 19. Hyperpolarized ^129^Xe MR has been shown in numerous studies to be a useful noninvasive probe of lung structure 20, 21, 22 and function 23, 24, 25, 26, 27, 28. Signal can be detected at low concentrations from this exogenous in vivo contrast agent, and owing to xenon's large, loosely bound electron cloud, ^129^Xe NMR is highly sensitive to the xenon chemical environment: 129Xe nuclei exhibit a marked change in resonance frequency when dissolved in different biological fluids and tissues 29, 30.

Three distinct NMR peaks are observed in vivo when ^129^Xe gas is inhaled into the lungs. The largest peak originates from ^129^Xe gas in the alveolar airspaces and the two other peaks, centered approximately 200 ppm away from the gas peak, have been attributed to ^129^Xe dissolved in lung parenchymal tissue/blood plasma (TP) and in red blood cells (RBCs) 31, 32. Physiologically important information about gas exchange can be derived from the signal amplitudes and exchange kinetics of these distinct ^129^Xe resonances 20, 33. Furthermore, the ^129^Xe resonance frequency in RBCs was shown previously by Wolber et al. to be sensitive to blood oxygen saturation, *s*O_2_ 34. Mechanisms underpinning the ^129^Xe chemical shift dependence on blood oxygenation are not yet fully understood, and the dependence is currently thought to be due to the conformational changes of hemoglobin as it binds and releases oxygen molecules 34.

In the study reported here, spectra of hyperpolarized ^129^Xe dissolved in samples of human blood were obtained to validate the previous in vitro work conducted at B_0_ = 1.5 T by Wolber et al. 34. In so doing, we determine the relationship between the ^129^Xe‐RBC resonance frequency and blood oxygenation, over the full oxygenation range at the two clinically relevant field strengths of 1.5 T and 3 T. Using this relationship, measurements of RBC *s*O_2_ in vivo in healthy human lungs were then derived using whole lung NMR spectroscopy measurements of lung oxygen desaturation during breath hold apnea. A technique for the noninvasive dynamic detection of RBC *s*O_2_ in vivo using hyperpolarized ^129^Xe is hereby demonstrated.

## METHODS

### Hyperpolarized ^129^Xe Gas Preparation

For in vitro and in vivo studies, a gas mixture of 3 % isotopically enriched xenon (86 % ^129^Xe), 10 % nitrogen and 87 % helium was flowed through a glass cell (volume 500 cm^3^; temperature 373 K; total gas pressure 2 bars) on a home‐built ^129^Xe spin‐exchange optical pumping polarizer 35 at a flow rate of 300 sccm (standard cubic centimeters per minute). Upon exiting the cell, the hyperpolarized ^129^Xe was cryogenically separated in a liquid nitrogen‐cooled distilator and collected in its frozen state over a time of ∼20 min (xenon volume ∼ 200 mL) for in vitro samples and ∼60 min (xenon volume 600 mL) for in vivo samples.

### Blood Sample Preparation and Analysis

Whole blood samples were withdrawn by a clinician from three self‐consenting healthy male volunteers (two Caucasian, ages 23 and 27; one Asian, age 35) by venipuncture and transferred into lithium heparin vacuum containers approximately 2–3 h before the start of the NMR experiments. All blood samples were allowed to equilibrate to a temperature of 20 ± 2 °C (the temperature at which the scanner room is maintained). Before conducting the NMR experiments, the xenon was first dissolved in the blood. To ensure effective mixing, the xenon and blood were passed through an exchange module 25, 36 (Superphobic MicroModule 0.5 X 1 G680 Contactor, Membrana, USA), which provided an exchange surface area of 100 cm^2^. The exchange module was also used to control the oxygenation of the blood samples before they were mixed with xenon.

A clinical blood gas analyzer (Radiometer, ABL80, UK) was used to analyze each blood sample and determine the following physiological parameters: *s*O_2_ (fraction of hemoglobin molecules that are fully oxygenated), *p*O_2_ (partial pressure of O_2_ in whole blood)_,_ *p*CO_2_ (partial pressure of CO_2_ in whole blood), HCT (fraction of red blood cells within whole blood) and pH. Immediately after acquiring NMR spectra as described below, approximately 0.1 mL of blood was withdrawn directly from the NMR sample syringe and taken to the blood gas analyzer for analysis. Full experimental details of the xenon/oxygen dissolution method and RBC *s*O_2_ measurement technique have been described previously in a study reporting the $T_{1}$ relaxation behavior of ^129^Xe in blood 37.

### In Vitro NMR Spectroscopy

A custom‐built, eight‐turn solenoid radiofrequency (RF) coil of 15 mm inner diameter was used for transmission/reception at the frequency of ^129^Xe dissolved in RBCs and plasma. The gas exchange membrane was positioned near the sensitive volume of the NMR coil to reduce the xenon‐blood transit time to the coil and, thus minimizing $T_{1}$ relaxation. To calibrate the excitation flip angle, a small sample of the hyperpolarized ^129^Xe‐blood mixture was placed within the active coil volume. Hard pulses of duration 500 μs were used to acquire spectra, and from these, the ^129^Xe‐RBC signal decrease was fitted to (cos *α*)*^n^* ^‐1^, where *α* is the flip angle to be calibrated and *n* is the RF pulse number 37. The excitation flip angles used were calculated to be in the range 10–15°.

**Figure 1:**
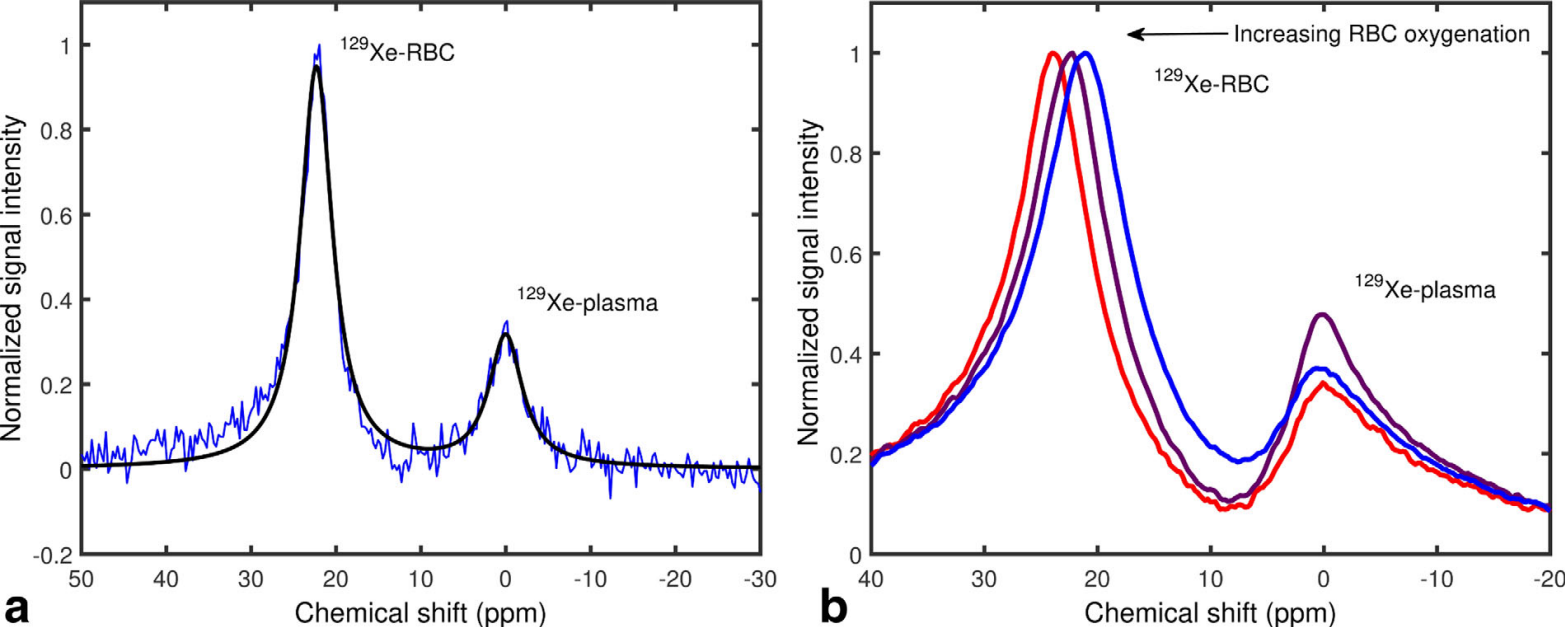
**a:** Single‐shot ^129^Xe‐blood spectrum at 1.5 T. The measured absorption spectrum (blue line) was fitted to a linear combination of two Lorentzians (Equation (1), solid black line) to determine peak positions. **b:** Example ^129^Xe‐blood spectra acquired at 3 T. With increasing oxygenation, the peak associated with ^129^Xe dissolved in RBCs is seen to shift measurably toward a higher resonance frequency.

Spectra were acquired on both 1.5 T (GE, HDx, USA) and 3 T (Philips, Achieva, Netherlands) MR scanners. At both *B*_0_ field strengths, 20 pulse‐acquire measurements were made with 512 sample points, a receiver bandwidth of 2.5 kHz and a repetition time (TR) of 500 ms. Free induction decay data were imported into MATLAB (R2011b, MathWorks, USA) for spectral analysis. Zero‐order phase corrections (on the ^129^Xe‐RBC resonance), followed by first‐order phase corrections (to phase the ^129^Xe‐plasma resonance, using the ^129^Xe‐RBC resonance as the zero frequency point) were performed on the averaged raw data, and absorption spectra, Re[*L*], were fitted in the chemical shift (*δ*) domain to a linear combination of two Lorentzians

(1) $$\text{Re}[L](\delta )\;=\;A_{r}\frac{\frac{1}{2}\Gamma_{r}}{{\left(\delta_{}-\delta_{r}^{0}\right)}^{2}+{\left(\frac{1}{2}\Gamma_{r}\right)}^{2}}+B_{p}\frac{\frac{1}{2}\Gamma_{p}}{{\left(\delta_{}-\delta_{p}^{0}\right)}^{2}+{\left(\frac{1}{2}\Gamma_{p}\right)}^{2}}$$

where $A_{r}$, $B_{p}$ are the amplitudes of the RBC and plasma ^129^Xe resonances; $\delta_{r}^{0}$, $\delta_{p}^{0}$ are the maxima of the ^129^Xe‐RBC and ^129^Xe‐plasma resonances; and $\Gamma_{r}$, $\Gamma_{p}$ are the full width half maxima of the two respective peaks. Figure 1a shows an example of a fit to a typical in vitro 1.5 T ^129^Xe‐blood spectrum.

### In Vivo NMR Spectroscopy

**Figure 2:**
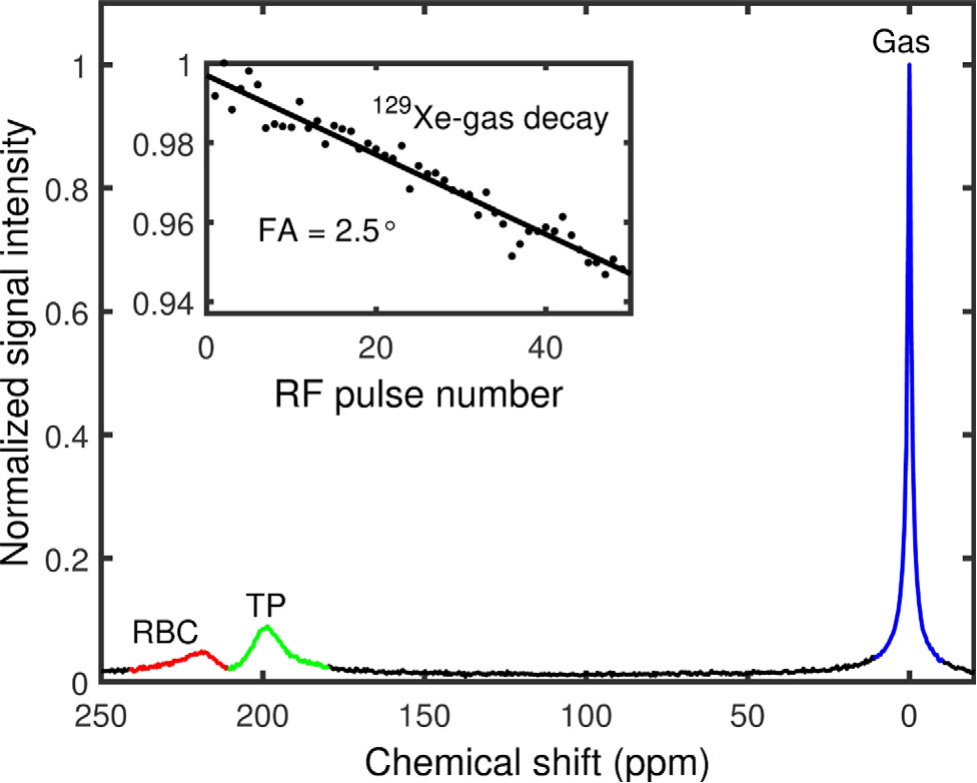
Magnitude NMR spectrum of ^129^Xe acquired from the human lungs after a hard pulse excitation centered ∼220 ppm away from the gas peak (depicted here as a large peak outlined in blue). Spectral peaks associated with parenchymal tissue/blood plasma (TP) and RBCs are an order of magnitude smaller and have broader linewidths (shorter *T*_2_*s) than the ^129^Xe‐gas peak. Inset: ^129^Xe‐gas resonance flip angle calibration for a hard pulse centered on the ^129^Xe‐dissolved resonance.

Whole‐lung spectroscopy experiments were then performed at 3 T (Philips, Achieva, Netherlands) on two healthy male Caucasian volunteers (24 and 28 years old). All experiments were performed during breath hold apnea lasting 35–40 s. A 360 μs duration 90° hard pulse centered ∼200 ppm downfield from the gaseous ^129^Xe resonance was used for excitation of the ^129^Xe dissolved in RBCs (^129^Xe‐RBC) and parenchymal tissue/blood plasma (^129^Xe‐TP) using a flexible quadrature transmit/receive RF coil tuned to 35.35 MHz (Clinical MR Solutions, USA). See Figure 2 for relative signal intensities corresponding to ^129^Xe‐gas, ^129^Xe‐RBC, and ^129^Xe‐TP peaks in the lungs measured at 3 T.

Two separate pulse‐acquire sequences with TRs of 100 ms (performed on the 28‐year‐old volunteer) and 800 ms (performed on both volunteers) were used, and the effective flip angle excitation on the ^129^Xe gas peak was determined to be ∼2.5° (see Figure 2) for a 90° excitation of the dissolved phase ^129^Xe. For the TR = 800 ms breath hold experiments, 2048 samples were acquired at a bandwidth of 3 kHz, corresponding to a spectral resolution of 1.46 Hz (0.04 ppm). To achieve a TR of 100 ms at 3 kHz bandwidth, the number of samples was decreased to 128, reducing the spectral resolution to ∼0.7 ppm. The spectra were phased and fitted to a double Lorentzian using the same method outlined in the previous section.

## RESULTS

### In Vitro Spectroscopy

The relationship between the chemical shift of ^129^Xe and *s*O_2_ of blood was calibrated in a controlled in vitro environment. The resonance frequency of ^129^Xe dissolved in plasma was used as a reference as it was found not to vary as a function of blood oxygenation in vitro. Increasing RBC *s*O_2_ caused an increase in the resonance frequency of ^129^Xe dissolved in RBCs. The RBC‐plasma peak separation increased from approximately 20.4 ppm, when the blood was in a completely deoxygenated state, to approximately 25.5 ppm when the blood was in a fully oxygenated state.

**Figure 3:**
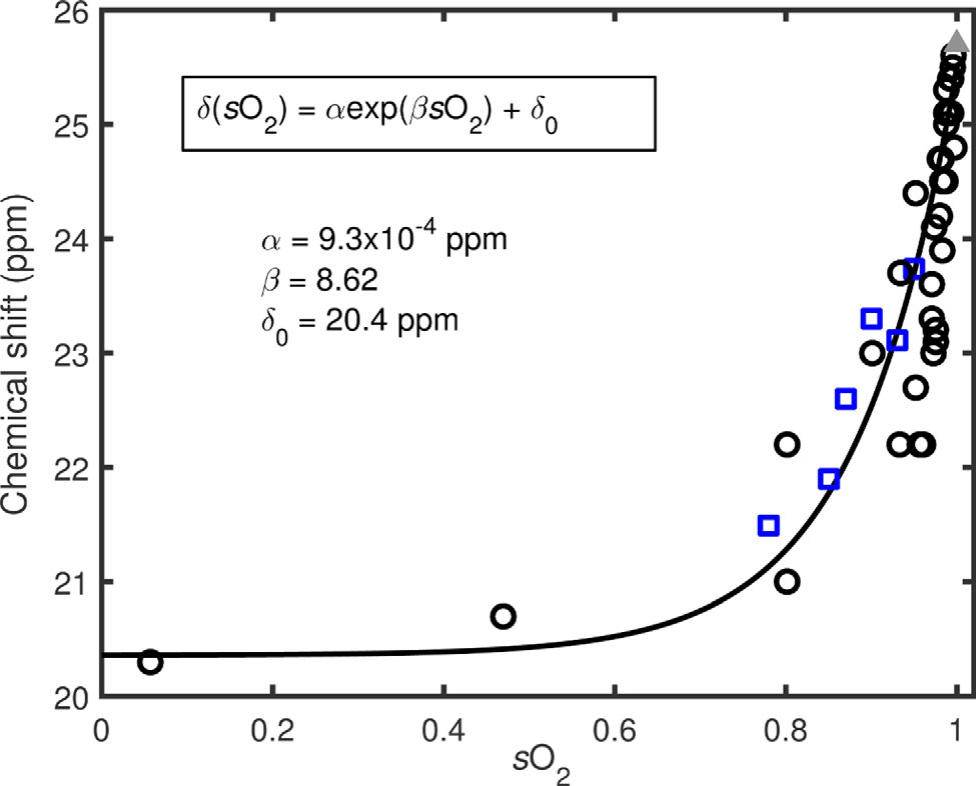
The ^129^Xe‐RBC chemical shift plotted against the measured blood oxygen saturation, *s*O_2_, with the ^129^Xe‐plasma peak as a 0 ppm reference peak. Data from the two field strengths are denoted by black open circles (1.5 T) and blue open squares (3 T). The black line is a fit to both the 1.5 T and 3 T data using Equation (2). The grey triangle represents blood equilibrated with carbon monoxide (simulating the fully oxygenated conformation of hemoglobin).

This observed chemical shift versus *s*O_2_ behavior was consistent at B_0_ magnetic field strengths of both 1.5 T and 3 T. To quantify the change in RBC‐plasma peak separation, δ, as a function of *s*O_2_, the ^129^Xe‐RBC and ^129^Xe‐plasma peak locations, $\delta_{r}^{0}$ and $\delta_{p}^{0}$, were determined by fitting the spectra to Equation (1). Blood gas analysis was performed immediately after acquiring the NMR spectra to quantify the RBC *s*O_2_. The extracted peak separations are plotted as a function of *s*O_2_ in Figure 3, where it can be observed that the chemical shift of ^129^Xe in RBCs appears to be nonlinearly dependent on the measured *s*O_2_, which is consistent with the previous observations of Wolber et al. 34. 1.5 T and 3 T data were fitted to a single empirical equation

(2) $$\delta (sO_{2})\;=\;\alpha \text{exp}(\beta sO_{2})+\delta_{0}$$

where, α and β are empirical constants and $\delta_{0}$ is the RBC‐plasma peak separation in fully deoxygenated blood (see Figure 3). The similarity in the measured ^129^Xe chemical shift values at 1.5 T and 3 T shown in Figure 3, therefore, suggest that the relationship between the ^129^Xe chemical shift and RBC *s*O_2_ is independent of the static magnetic field strength.

### In Vivo Detection of Chemical Shift

**Figure 4:**
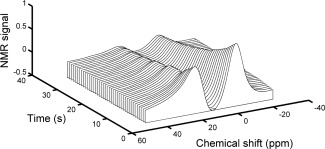
Waterfall plot of a typical time series of in vivo ^129^Xe‐dissolved spectra. The resonance at 0 ppm is from ^129^Xe dissolved in parenchymal parenchymal tissue/blood plasma (TP) and the resonance downfield is from ^129^Xe dissolved in RBCs. Each spectrum was acquired using a flip angle of 90° and a TR of 800 ms. The decay in both compartments follows approximately the *T*_1_ relaxation (due to the presence of oxygen in the lungs and RF excitation) of the ^129^Xe‐gas, the magnetization reservoir that acts to replenish the ^129^Xe‐dissolved signal between RF pulses.

To determine whether a similar shift in the resonance frequency of ^129^Xe in RBCs could be detected in vivo as a potential noninvasive probe for pulmonary blood oxygenation, a series of NMR spectra were acquired during breath hold apnea, which provides a simple model for blood oxygenation change. Figure 4 shows a waterfall plot of representative dissolved ^129^Xe spectra acquired as a function of time during one such experiment. The SNR of the spectrum at the beginning of the acquisition series was measured to be ∼50, dropping to ∼7 after 35 s of breath hold apnea. For these typical SNRs, it was possible to perform good Lorentzian fits on the data, enabling accurate peak assignment. The ^129^Xe resonance located at 0 ppm corresponds to the ^129^Xe‐TP resonance and the resonance located downfield is from ^129^Xe dissolved in RBCs. For all datasets, an initial rapid drop in the ^129^Xe‐RBC signal was observed, which we believe to be a result of RF‐induced depolarization of the postcapillary signal from the ^129^Xe in the pulmonary veins for TRs shorter than (TR = 100 ms), or of the order of (TR = 800 ms), the RBC capillary transit time of ∼750 ms 38.

**Figure 5:**
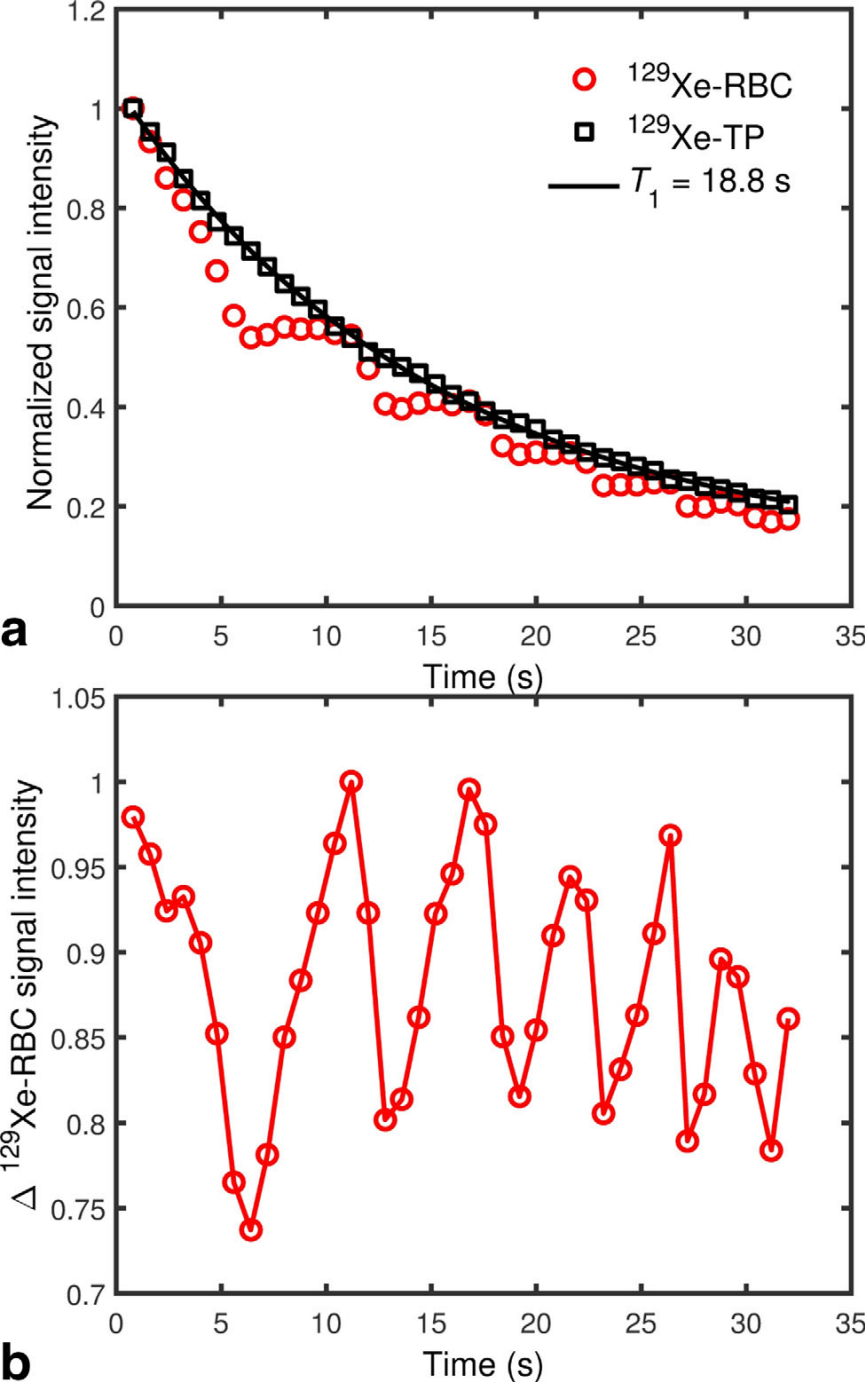
**a:** Decaying in vivo ^129^Xe‐dissolved signal in human lungs during breath‐hold apnea. The red circles and black squares represent ^129^Xe in RBCs and parenchymal tissue/blood plasma (TP), respectively. While the ^129^Xe‐TP signal decays monotonically, the ^129^Xe‐RBC signal does not. The black line is an exponential fit to the decaying ^129^Xe‐TP signal, from which a ^129^Xe‐gas $T_{1}$ was estimated. **b:** ^129^Xe‐RBC signal divided by the fitted exponential in (a), expressed as a fractional change (Δ) in signal intensity.

For TR = 800 ms, the ^129^Xe‐TP signal exhibited a monotonic decay over the breath hold time (Figure 5a). It was assumed that this signal decay follows the $T_{1}$ of the ^129^Xe gas (dominated by dipolar coupling of the nuclear spin with paramagnetic oxygen in the lungs), which acts as a longitudinal magnetization reservoir, replenishing the ^129^Xe‐dissolved magnetization in between RF pulses. With this assumption, a $T_{1}$ fit (corrected for ^129^Xe‐gas signal decay due to off‐resonance RF excitations of 2.5° on the gas resonance, which result from the side bands of the hard RF pulses used) was performed on the decaying ^129^Xe‐TP signal, resulting in an approximate ^129^Xe‐gas $T_{1}$ value of 18.8 s, which is in good agreement with previously measured ^129^Xe‐gas $T_{1}$ values of ∼ 20 s 39. Although the ^129^Xe‐RBC signal was observed to decay with the same overall rate as the ^129^Xe‐TP signal, it did not decay monotonically; instead, the signal was observed to modulate periodically over the breath hold time (see Figure 5a).

To view this oscillatory decay behavior more clearly, the ^129^Xe‐RBC signal was normalized to the $T_{1}$ decay of the ^129^Xe‐gas polarization reservoir (^129^Xe‐RBC data were divided pointwise by the fitted $T_{1}$ curve – see Figure 5b). For both volunteers, the ^129^Xe‐RBC peak‐to‐peak signal interval (i.e. modulation period) was measured to be 4–8 s (0.125–0.25 Hz).

**Figure 6:**
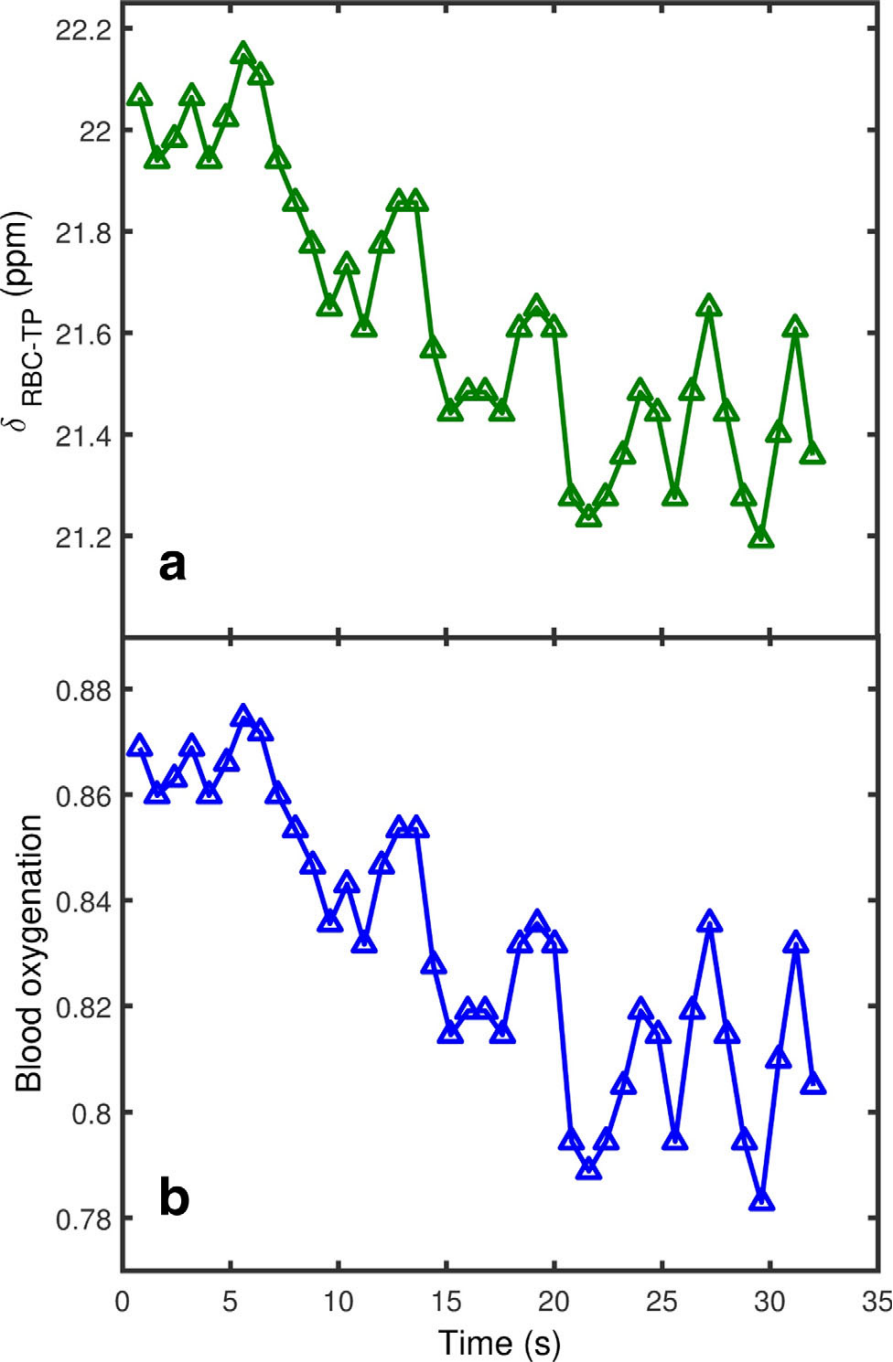
**a:** ^129^Xe‐RBC chemical shift in human lungs in vivo over the course of a breath hold, where a clear periodic modulation is observed. **b:** Calculated blood oxygenation over the breath hold, derived from the measured ^129^Xe‐RBC chemical shifts using Equation (2).

Whereas the ^129^Xe‐TP resonance was observed to remain fixed in position over the 35 s breath hold, the ^129^Xe‐RBC resonance chemical shift was observed to decrease by approximately 1 ppm. The first observation of in vivo ^129^Xe‐RBC resonance shifts with lung oxygenation was first reported in our preliminary work 40, and these findings have been recently confirmed by Kaushik et al. 41, who observed a decrease in the ^129^Xe‐RBC resonance frequency in patients with idiopathic pulmonary fibrosis relative to healthy normals. Here, the ^129^Xe‐RBC chemical shift exhibited a periodic modulation at the same frequency as the ^129^Xe‐RBC signal oscillation, with a 180° phase difference (Figs. 6a, 8). Equation (2) was used to determine *s*O_2_ values from the measured in vivo ^129^Xe‐RBC chemical shift values. The *s*O_2_ in both volunteers at the start of the breath hold was measured to be ∼0.87, dropping to ∼0.80 at the end of the 35 s breath hold (Figure 6b).

In these calculations, it was assumed that the temperature difference in vitro (20 °C) and in vivo (37 °C) has a negligible effect on the ^129^Xe‐RBC and ^129^Xe‐TP resonances, i.e. the calibration curve in Figure 3 is valid for converting the in vivo ^129^Xe‐RBC chemical shift to pulmonary *s*O_2_. To test the validity of this assumption, NMR spectra of ^129^Xe dissolved in plasma were acquired at 20 °C and 37 °C with a ^129^Xe gas compartment as a 0 ppm reference. No differences in the ^129^Xe‐gas and ^129^Xe‐plasma resonances were observed when the sample was heated from 20 °C to 37 °C. Albert et al. 42 previously measured a chemical shift difference of ∼20 ppm between the ^129^Xe‐RBC and ^129^Xe‐plasma resonances in deoxygenated blood at a temperature of 8 °C, which is in good agreement with the chemical difference of 20.4 ppm we have observed at 20 °C. We therefore assume the ^129^Xe‐RBC resonance is independent of temperature over the range 20 °C to 37 °C. For TR = 100 ms, both the ^129^Xe‐TP and ^129^Xe‐RBC signals exhibited oscillations with an approximately constant peak‐to‐peak signal interval (modulation period) of ∼1 s over the breath hold duration.

## DISCUSSION

### In Vitro Spectroscopy

The observation of a nonlinear dependence of ^129^Xe‐RBC chemical shift on *s*O_2_ has been reported in previous work by Wolber et al. 34, where it was concluded that bulk magnetic susceptibility differences between oxyhemoglobin (diamagnetic) and deoxyhemoglobin (paramagnetic) are not responsible for the ∼5 ppm ^129^Xe‐RBC chemical shift difference between oxygenated and deoxygenated blood. Instead, the authors hypothesized that the observed nonlinear relationship between blood oxygenation and chemical shift is the result of an oxygen‐dependent hemoglobin affinity for xenon. To date, a total of 12 xenon binding sites have been located in deoxyhemoglobin using X‐ray crystallography 43. To our knowledge, the binding sites of xenon in oxyhemoglobin have not been reported, making a quantitative description of the chemical shift mechanisms responsible for the observed nonlinear change in chemical shift with blood of oxygenation difficult at this time. Nevertheless, herein, we draw on the insights of Wolber et al. 34 to further understand the mechanisms governing the observed nonlinear relationship between the ^129^Xe‐RBC chemical shift and blood oxygenation.

The resonance frequency of ^129^Xe in a solvent solution is determined by the local magnetic field experienced at the nucleus, which is mediated by the screening (or shielding) constant, *σ*:

(3) $$\omega_{Obs}\;=\;\gamma B_{eff}$$

(4) $$B_{eff}\;=\;(1-\sigma )B_{0}$$

(5) $$\omega_{Obs}\;=\;\gamma (1-\sigma )B_{0}$$

where $\omega_{Obs}$ is the observed resonance frequency, γ is the gyromagnetic ratio, $B_{0}$ is the applied static magnetic field and $B_{eff}$ is the effective field at the nucleus. Following the pioneering work of Buckingham et al. 44, it is known that the screening constant, σ, of a nucleus can be expressed as the sum of the screening constant of the individual nucleus, $\sigma^{0}$, and a term arising due to the presence of the solvent medium, $\sigma_{m}$, thus

(6) $$\sigma \;=\;\sigma^{0}+\sigma_{m}$$

(7) $$\sigma_{m}\;=\;\sigma_{a}+\sigma_{e}+\sigma_{w}+\sigma_{b}$$

where $\sigma_{a}$ is a contribution from molecular anisotropy effects, $\sigma_{e}$ is the polar effect caused by an electric field, $\sigma_{w}$ is due to the van der Waals forces between the solute and solvent and $\sigma_{b}$ indicates the shielding arising from bulk magnetic susceptibility effects within the solvent, which we neglect as a significant contributor to ^129^Xe chemical shift changes with blood oxygenation from the conclusions drawn by Wolber et al. 34. For ^129^Xe dissolved in solution at 20 °C and at body temperature (37 °C), the anisotropic and electric field terms are zero 45, leaving only the van der Waals shielding term.

Stephen 46 has demonstrated that the van der Waals deshielding experienced by a nucleus within a solvent can be expressed as $-\sigma_{w}\;=\;B{\bar{F}}^{2}$, where ${\bar{F}}^{2}$ is the mean square electric field brought about by fluctuations among electrons located on the neighboring solvent molecules and *B* is the “shielding hyperpolarizability” 47, which is significant for ^129^Xe, whose nucleus is surrounded by a large, easily‐deformed electron cloud. As a result of this high shielding hyperpolarizabilty, the chemical shift of dissolved ^129^Xe in a solvent is very sensitive to small differences in the dispersion fields acting within liquid solvents. The most commonly used approach to correlate solvent‐induced ^129^Xe chemical shifts with ${\bar{F}}^{2}$ is to use a continuum model to describe the solvent 48, 49. Specifically, ${\bar{F}}^{2}$ is proportional to the square of the Bayliss‐McRae function, g(n) 49, i.e., ${\bar{F}}^{2}\propto {\left[g(n)\right]}^{2}\;=\;{\left[(n^{2}-1)/(2n^{2}+1)\right]}^{2}$, where *n* is the index of refraction of the solvent. The refractive indices of oxyhemoglobin and deoxyhemoglobin have been recently measured by Zhernovaya et al. 50, wherein no significant difference between the refractive index of deoxygenated and oxygenated hemoglobin (within the visible range of the spectrum) was reported. We therefore conclude that the observed ^129^Xe‐RBC chemical shift change with blood oxygenation cannot be predicted using whole blood refractive index measurements.

Experiments using Mössbauer spectroscopy 51 suggest that the electron cloud of hemoglobin is drawn toward the highly electronegative oxygen molecule in oxyhemoglobin, but is more evenly distributed in deoxyhemoglobin. Similarly, as ^129^Xe forms transient van der Waals bonds with hemoglobin, the net electron cloud of ^129^Xe is likely to be drawn to the more electronegative O_2_ molecule in oxyhemoglobin. This would act to increase the deshielding of the ^129^Xe nucleus, thereby reducing $\sigma_{w}$, resulting in an increased ^129^Xe‐RBC resonance frequency, which is in agreement with the observed data.

In addition to changes in the electronegativity of the hemoglobin molecules with blood oxygenation, the xenon‐hemoglobin binding site locations may change as the hemoglobin makes transitions between oxy‐ and deoxy‐conformations. As the extent of the shielding constant $\sigma_{w}$ is strongly dependent on the separation between a nucleus and the molecules giving rise to fluctuating electric fields 47, changes in position would greatly alter the magnitude of the mean square field, ${\bar{F}}^{2}$, experienced by the ^129^Xe nucleus within the hemoglobin. Each RBC contains a large number of individual hemoglobin molecules, and the fraction of hemoglobin molecules in the oxy‐ and deoxy‐conformations has been measured previously in horse hemoglobin to vary smoothly as a function of blood oxygenation 52. The intracellular environment experienced by ^129^Xe nuclei would thus be expected to vary smoothly as a function of blood oxygenation, which is consistent with the observation of a smooth nonlinear change in the ^129^Xe‐RBC resonance with blood oxygenation.

**Figure 7:**
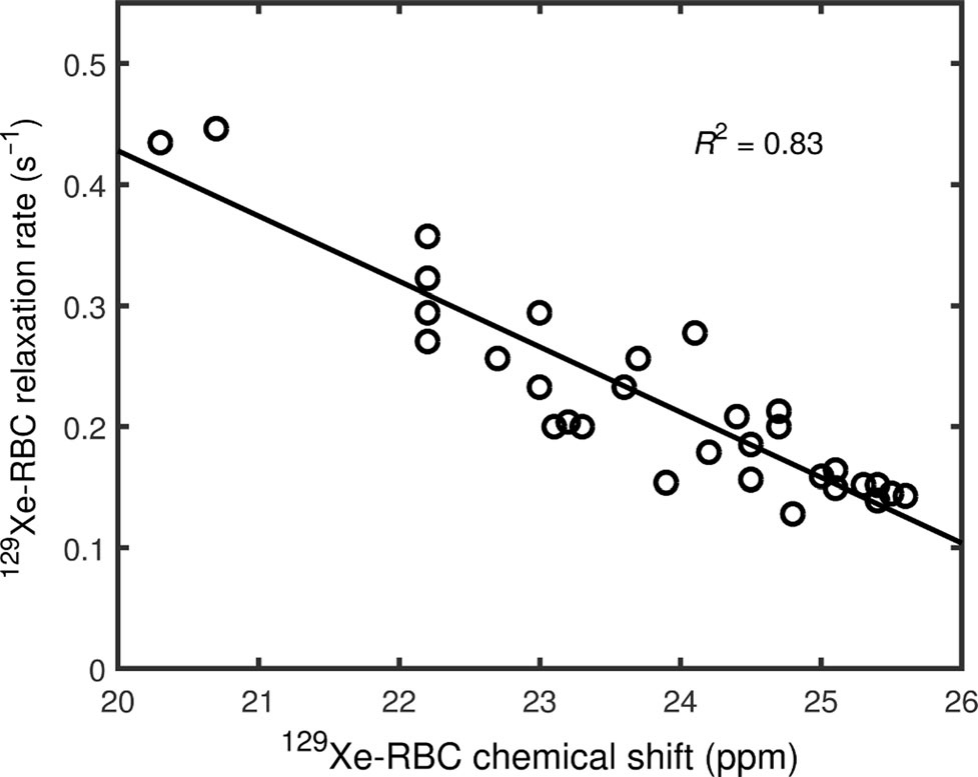
Correlation of ^129^Xe‐RBC relaxation rate and ^129^Xe‐RBC chemical shift in whole blood samples over the full (0.06–1.00) blood oxygenation range.

Finally, to help determine whether the underlying mechanisms driving the change in ^129^Xe relaxation rate and ^129^Xe‐RBC chemical shift with *s*O_2_ are related, a correlation plot of the variation of the two NMR parameters was generated, as shown in Figure 7. The ^129^Xe‐RBC relaxation rate data are taken from our previous study 37 in which the relationship between ^129^Xe‐RBC relaxation rate and *s*O_2_ was determined. The plot reveals that changes in ^129^Xe‐RBC relaxation rate and ^129^Xe‐RBC chemical shift are well correlated, with a coefficient of determination of *R* ^2^ = 0.83. This presents strong empirical evidence that the underlying physical mechanism driving these observed changes in chemical shift and relaxation rate is the same. As the chemical shift and relaxation rate of ^129^Xe in solvents is strongly dependent on intermolecular separations, we therefore conclude it is likely that oxygen‐modulated xenon binding is largely responsible for the observed ^129^Xe‐RBC chemical shift and relaxation rate *s*O_2_ dependencies.

### In Vivo Spectroscopy

**Figure 8:**
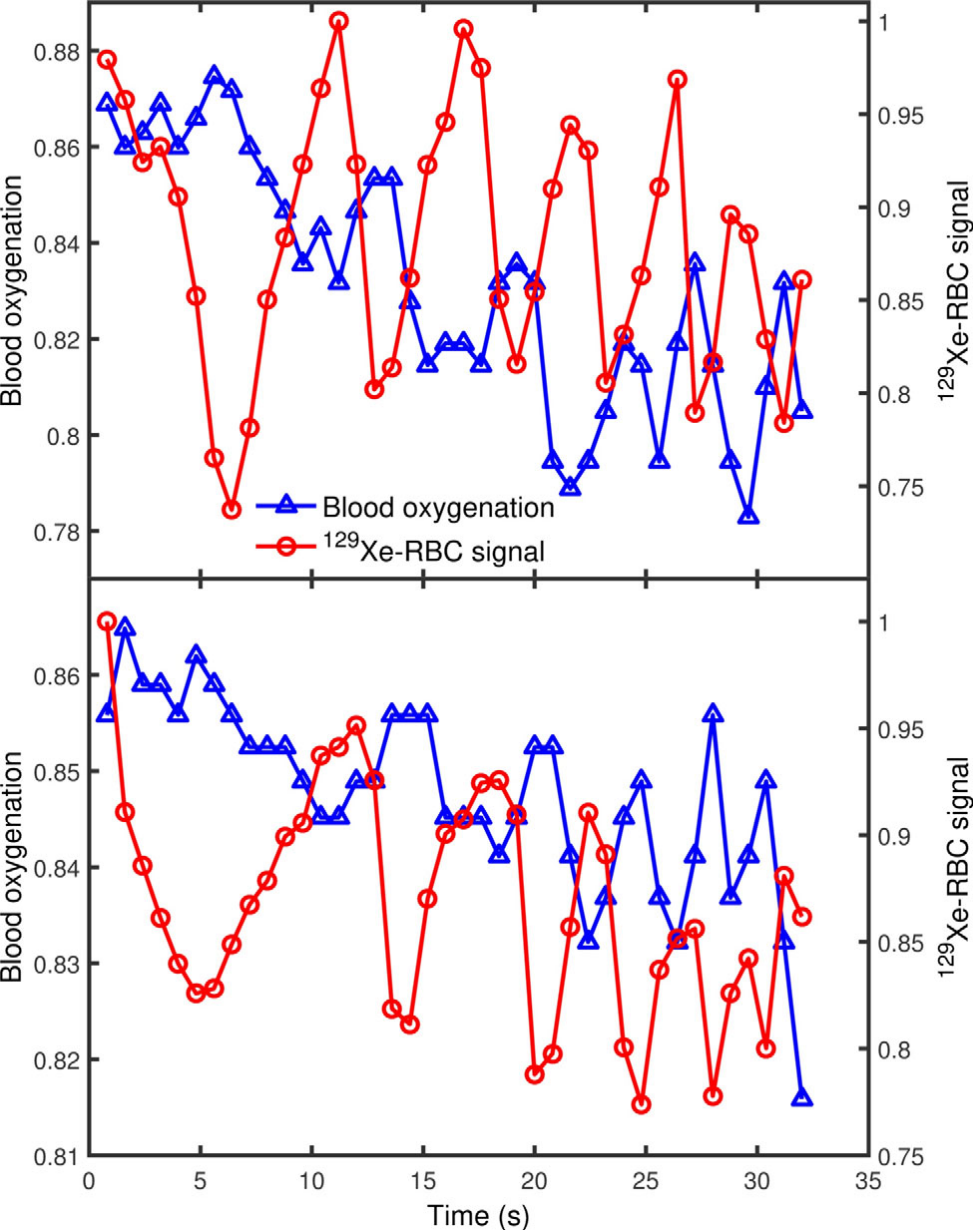
In vivo lung blood oxygenation and ^129^Xe‐RBC signal changes over breath hold apnea measured from two healthy volunteers, (**a**) and (**b**) (28 and 24 years old, respectively). The blood oxygenation and ^129^Xe‐RBC signal oscillate at the same frequency where the blood oxygenation maxima/minima coincide with ^129^Xe‐RBC signal minima/maxima (i.e. a phase difference of 180°).

As shown in Figures 8a and b, for TR = 800 ms, the *s*O_2_ values calculated from the ^129^Xe‐RBC chemical shift were shown to oscillate over the breath hold at the same frequency as the ^129^Xe‐RBC signal amplitude modulation, but with a 180° phase difference; the *s*O_2_ maxima were observed to coincide with the ^129^Xe‐RBC signal minima, suggestive of a link between pulmonary oxygenation and ^129^Xe‐RBC signal changes. The calculated *s*O_2_ of ∼0.87 for both volunteers at the beginning of the breath hold suggests that the observed ^129^Xe‐RBC signal comprises ^129^Xe dissolved in blood containing RBCs with mixed *s*O_2_ values; otherwise it would be expected that the calculated *s*O_2_ would equal either 1.00 for fully oxygenated blood or ∼0.75 for deoxygenated blood. Indeed, for TR values of 100–800 ms, the observed ^129^Xe‐RBC signal should be constituted primarily by ^129^Xe nuclei dissolved in blood circulating in the alveolar capillary bed, which contains blood with a range of *s*O_2_ values. Signal contribution from ^129^Xe dissolved in blood circulating in the pulmonary arteries (precapillary blood) is assumed to be negligible as a result of ^129^Xe polarization losses incurred during ^129^Xe transit in the systemic circulation. Signal from ^129^Xe in the pulmonary veins is also considered to be negligible, as the postcapillary ^129^Xe signal is destroyed by 90° RF pulses for TR values of 100–800 ms, which are of the order of the RBC capillary transit time of 750 ms.

A first‐order estimate of the expected average *s*O_2_ of RBCs which contribute to the observed ^129^Xe‐RBC signal for TR = 800 ms can be derived using the following assumption: the average RBC oxygenation in the capillaries *s*O_2_c = [0.75 (deoxygenated) + 1.00 (oxygenated)] / 2 = 0.88. This agrees well with the *s*O_2_ value of 0.87 calculated from the ∼22 ppm chemical shift observed at the start of breath hold apnea in both healthy volunteers for TR = 800 ms.

After 35 s of breath hold apnea, a decrease in the observed $sO_{2}$ of 7–10 % was calculated for both volunteers. During breath hold the oxygen partial pressure, *p*O_2_, in the lungs will decrease over time. Measurements of changes in oxygen partial pressure as a function of breath hold have been performed in both animal and human lungs using hyperpolarized ^3^He MR 16, 53, 54. It was shown in humans that over short breath holds (< 40 s), the decrease in *p*O_2_ can be approximated by a linear relationship 15, 16, 53

(8) $$pO_{2}(t)\;=\;p_{0}-Rt$$

where *R* is the rate of oxygen extraction by perfusion and $p_{0}$ is the initial *p*O_2_. Previous studies involving *p*O_2_ mapping with ^3^He in healthy volunteers 16, 53, 55 reported *R* to decrease with decreasing $p_{0}$. This may be a result of the lower baseline *p*O_2_ reducing the alveolar‐capillary *p*O_2_ gradient, and, therefore, decreasing the rate of diffusion of oxygen from the alveoli into the capillaries. It is worth noting that over long breath holds and/or large *R* values, an exponential model of oxygen depletion should be used instead of Equation (8), which for small *t* is the first term in the Taylor expansion of an exponential function 54. Assuming a functional residual capacity of 3 L for the healthy male volunteers in this study and a resting *p*O_2_ of 140 mbar 38, the *p*O_2_ drop upon inhalation of 1 L of anoxic gas (Xe/N_2_) can be estimated by scaling the *p*O_2_ with the increase in lung volume to 4 L, i.e. $p_{0}\;=\;140\times (3/4)\approx 105$ mbar. The *R* value for $p_{0}$= 105 mbar has been reported to be *R* = 0.7 mbar/s 55. Inserting these values into Equation (8), the *p*O_2_ is estimated to drop to *p*O_2_ = 81 mbar at the end of 35 s of breath hold apnea, which corresponds to a saturation *s*O_2_ value of 0.89 from the standard oxygen‐hemoglobin dissociation curve 56, 57. Using this value, the average alveolar capillary bed oxygenation can be calculated as *s*O_2_c = [0.75 (deoxygenated) + 0.89 (oxygenated)] / 2 = 0.82, which is in reasonable agreement with the *s*O_2_ estimates (measured from the ^129^Xe chemical shift) of approximately 0.80 for both volunteers after ∼35 s of breath hold apnea.

**Figure 9:**
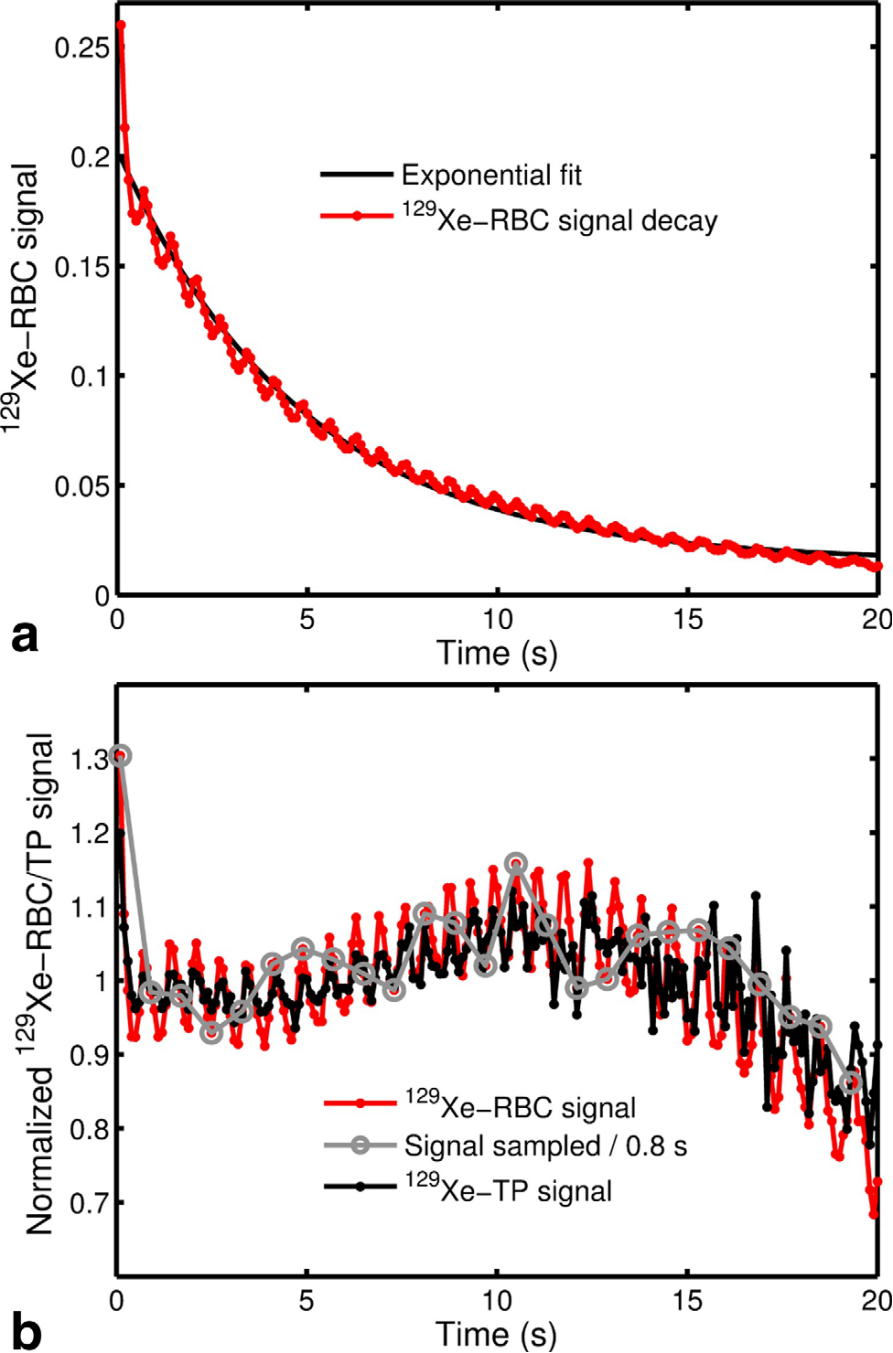
**a:** In vivo ^129^Xe‐RBC signal in the lungs as a function of breathhold time for a TR of 100 ms, fitted with an exponential function. **b:** In vivo ^129^Xe‐RBC and ^129^Xe‐TP signal changes normalized to the fitted exponential in (a). The gray line illustrates the effect of sampling the observed ^129^Xe‐RBC and ^129^Xe‐TP signal oscillations at a rate of 800 ms^−1^.

For TR = 100 ms, both the ^129^Xe‐RBC and ^129^Xe‐TP signals were observed to oscillate at a frequency close to the cardiac pulsation frequency (Figure 9), suggesting that the lower frequency signal oscillation observed for TR = 800 ms (Figure 8) is actually an alias of the higher frequency oscillation observed for TR = 100 ms. The gray line in Figure 9b shows artificial signal sampling at a rate of 800 ms^−1^ to illustrate this effect. ^129^Xe‐RBC and ^129^Xe‐TP signal oscillations at similar cardiac pulsation frequencies were first observed by Venkatesh et al. 58 and have been more recently observed by Ruppert et al. 59, where the signal oscillations were attributed to changes in blood flux into the capillaries within the cardiac cycle.

For the faster‐sampled experiment at TR = 100 ms, the spectral resolution was limited to 0.7 ppm, which was insufficient to spectrally discriminate changes in the ^129^Xe‐RBC chemical shift over the breath hold. It is possible that the observed *s*O_2_ for TR = 100 ms would be lower than that observed for TR = 800 ms; for short TRs, the RBCs have less time to travel through the capillaries into the pulmonary veins in between RF pulses (i.e. it is possible that the observable pulmonary *s*O_2_ varies as a function of the TR used). Cardiac‐gated acquisitions may allow the probing of ^129^Xe‐RBC chemical shifts (*s*O_2_) at specific time points in the cardiac cycle, opening up the possibility of using ^129^Xe NMR to quantify temporal blood oxygenation changes in the cardiopulmonary vascular circuit.

With the assumption that the ^129^Xe‐TP signal modulation arises from blood plasma flux changes within the cardiac cycle, it is possible to compare the peak‐to‐peak ^129^Xe‐RBC signal, amplitude, S_A_, with the ^129^Xe‐TP signal amplitude, S_B_, to estimate the relative RBC and plasma concentrations in the blood circulating through the capillaries. Thus, by taking the average of the first five S_A_ and S_B_ values after steady‐state was reached, $s_{a}\;=\;0.113\pm 0.004$ and $s_{b}\;=\;0.050\pm 0.003$, respectively, the HCT in the capillaries may be estimated from

(9) $$H\text{C}{\text{T}}_{\text{c}}\;=\;\frac{x}{x+1}$$

where $x\;=\;S_{a}\delta_{b}/S_{b}\delta_{a}$ and $\delta_{a}$= 0.27, $\delta_{b}$= 0.094 are the solubilities of xenon in RBCs and plasma at 37 °C 60. Inserting these values into Equation (9) gives HCT_c_ = 0.44, in good agreement with the Fåhræus effect which predicts HCT < 0.50 61 for blood flowing in narrow vessels such as the pulmonary capillaries. This is potentially of interest clinically; however, further work is required to validate this technique for measurement of HCT in the pulmonary capillaries.

This dissolved ^129^Xe spectroscopy oximetry technique may also have applications outside of the lungs. For example, Mazzanti et al. 62 have recently shown that the signal of ^129^Xe dissolved in the rat brain can be modulated in a manner responsive to stimuli, suggestive that there is a significant contribution to the measured ^129^Xe signal from blood flow/perfusion. Moreover, dissolved ^129^Xe spectra from the human brain have been successfully acquired 63, suggesting that ^129^Xe can be detected in organs quite distal from the point of uptake in the lungs.

Finally, this work has implications for Dixon‐based dissolved ^129^Xe imaging methods 64, 65, which rely upon a fixed chemical shift difference between the target spectroscopic compartments; the frequency difference between compartments is encoded as a phase shift in a given echo time and the different echo times need to be fixed in value. Therefore, drift in the ^129^Xe‐RBC peak position would affect the intensity of the ^129^Xe‐RBC phase image.

## CONCLUSIONS

In this study, the feasibility of using hyperpolarized ^129^Xe as an exogenous NMR probe of pulmonary blood oxygenation in humans has been explored. A nonlinear relationship between the measured *s*O_2_ values and the NMR resonance frequency of ^129^Xe dissolved in RBCs has been observed in vitro from blood samples at 1.5 T and 3 T. This relationship was evaluated over the entire range of possible blood oxygenation values and appears to be independent of magnetic field strength.

Furthermore, this relation has been used to derive lung blood *s*O_2_ values by means of in vivo dissolved ^129^Xe whole‐lung spectroscopy experiments conducted during apnea on a 3 T whole body system. To date, we are not aware of any other means of noninvasively measuring pulmonary blood oxygenation. The common modulation frequency of blood oxygenation and ^129^Xe‐RBC signal change during breath hold is interesting and further work with cardiac‐gated hyperpolarized ^129^Xe NMR is underway to help understand this newly observed phenomenon. Lastly, the HCT in the pulmonary capillaries has been estimated from ^129^Xe spectroscopic data. Further work is underway to validate hyperpolarized ^129^Xe NMR as a noninvasive technique for quantification of HCT in the pulmonary capillaries.

## References

[1] Astrup J , Siesjo BK , Symon L . Thresholds in cerebral ischemia ‐ the ischemic penumbra. Stroke 1981;12:723–725. 6272455 10.1161/01.str.12.6.723

[2] Rahimtoola SH . The hibernating myocardium. Am Heart J 1989;117:211–221. 2783527 10.1016/0002-8703(89)90685-6

[3] Thomlinson RH , Gray LH . The histological structure of some human lung cancers and the possible implications for radiotherapy. Br J Cancer 1955;9:539–549. 13304213 10.1038/bjc.1955.55 PMC2073776

[4] Hoenderdos K , Condliffe A . The neutrophil in chronic obstructive pulmonary disease. Am J Respir Cell Mol Biol 2013;48:531–539. 23328639 10.1165/rcmb.2012-0492TR

[5] Kreuzer F , Nessler CG Jr . Method of polarographic in vivo continuous recording of blood oxygen tension. Science 1958;128:1005–1006. 13592285 10.1126/science.128.3330.1005

[6] Chieregato A , Targa L , Zatelli R . Limitations of jugular bulb oxyhemoglobin saturation without intracranial pressure monitoring in subarachnoid hemorrhage. J Neurosurg Anesthesiol 1996;8:21–25. 8719188 10.1097/00008506-199601000-00006

[7] Kidwell CS , Alger JR , Saver JL . Beyond mismatch: evolving paradigms in imaging the ischemic penumbra with multimodal magnetic resonance imaging. Stroke 2003;34:2729–2735. 14576370 10.1161/01.STR.0000097608.38779.CC

[8] Dunn JF , O'Hara JA , Zaim‐Wadghiri Y , Lei H , Meyerand ME , Grinberg OY , Hou H , Hoopes PJ , Demidenko E , Swartz HM . Changes in oxygenation of intracranial tumors with carbogen: a BOLD MRI and EPR oximetry study. J Magn Reson Imaging 2002;16:511–521. 12412027 10.1002/jmri.10192

[9] Ogawa S , Lee TM , Kay AR , Tank DW . Brain magnetic resonance imaging with contrast dependent on blood oxygenation. Proc Natl Acad Sci U S A 1990;87:9868–9872. 2124706 10.1073/pnas.87.24.9868 PMC55275

[10] Taylor NJ , Baddeley H , Goodchild KA , et al. BOLD MRI of human tumor oxygenation during carbogen breathing. J Magn Reson Imaging 2001;14:156–163. 11477674 10.1002/jmri.1166

[11] Baudelet C , Gallez B . How does blood oxygen level‐dependent (BOLD) contrast correlate with oxygen partial pressure (pO2) inside tumors? Magn Reson Med 2002;48:980–986. 12465107 10.1002/mrm.10318

[12] Matsumoto S , Yasui H , Batra S , et al. Simultaneous imaging of tumor oxygenation and microvascular permeability using Overhauser enhanced MRI. Proc Natl Acad Sci U S A 2009;106:17898–17903. 19815528 10.1073/pnas.0908447106 PMC2761243

[13] Krishna MC , English S , Yamada K , et al. Overhauser enhanced magnetic resonance imaging for tumor oximetry: coregistration of tumor anatomy and tissue oxygen concentration. Proc Natl Acad Sci U S A 2002;99:2216–2221. 11854518 10.1073/pnas.042671399 PMC122345

[14] Hunjan S , Zhao D , Constantinescu A , Hahn EW , Antich PP , Mason RP . Tumor oximetry: demonstration of an enhanced dynamic mapping procedure using fluorine‐19 echo planar magnetic resonance imaging in the Dunning prostate R3327‐AT1 rat tumor. Int J Radiat Oncol Biol Phys 2001;49:1097–1108. 11240252 10.1016/s0360-3016(00)01460-7

[15] Deninger AJ , Eberle B , Ebert M , et al. Quantification of regional intrapulmonary oxygen partial pressure evolution during apnea by 3He MRI. J Magn Reson 1999;141:207–216. 10579944 10.1006/jmre.1999.1902

[16] Wild JM , Fichele S , Woodhouse N , Paley MNJ , Kasuboski L , van Beek EJR . 3D volume‐localized pO2 measurement in the human lung with 3He MRI. Magn Reson Med 2005;53:1055–1064. 15844148 10.1002/mrm.20423

[17] Sherriff SB , Smart RC , Taylor I . Clinical study of liver blood flow in man measured by 133Xe clearance after portal vein injection. Gut 1977;18:1027–1031. 606629 10.1136/gut.18.12.1027 PMC1411835

[18] Latchaw RE , Yonas H , Pentheny SL , Gur D . Adverse reactions to xenon‐enhanced CT cerebral blood flow determination. Radiology 1987;163:251–254. 3823444 10.1148/radiology.163.1.3823444

[19] Driehuys B , Martinez‐Jimenez S , Cleveland ZI , et al. Chronic obstructive pulmonary disease: safety and tolerability of hyperpolarized 129Xe MR imaging in healthy volunteers and patients. Radiology 2012;262:279–289. 22056683 10.1148/radiol.11102172 PMC3244666

[20] Patz S , Muradian I , Hrovat MI , Ruset IC , Topulos G , Covrig SD , Frederick E , Hatabu H , Hersman FW , Butler JP . Human pulmonary imaging and spectroscopy with hyperpolarized 129Xe at 0.2T. Acad Radiol 2008;15:713–727. 18486008 10.1016/j.acra.2008.01.008 PMC2475597

[21] Stewart NJ , Norquay G , Griffiths PD , Wild JM . Feasibility of human lung ventilation imaging using highly polarized naturally abundant xenon and optimized three‐dimensional steady‐state free precession. Magn Reson Med 2015;74:346–352. 25916276 10.1002/mrm.25732

[22] Kaushik SS , Cleveland ZI , Cofer GP , et al. Diffusion‐weighted hyperpolarized 129Xe MRI in healthy volunteers and subjects with chronic obstructive pulmonary disease. Magn Reson Med 2011;65:1154–1165. 21413080 10.1002/mrm.22697 PMC3351270

[23] Ruppert K , Brookeman JR , Hagspiel KD , Mugler JP . Probing lung physiology with xenon polarization transfer contrast (XTC). Magn Reson Med 2000;44:349–357. 10975884 10.1002/1522-2594(200009)44:3<349::aid-mrm2>3.0.co;2-j

[24] Patz S , Muradyan I , Hrovat MI , Dabaghyan M , Washko GR , Hatabu H , Butler JP . Diffusion of hyperpolarized 129Xe in the lung: a simplified model of 129Xe septal uptake and experimental results. New J Phys 2011;13:015009.

[25] Cleveland ZI , Moller HE , Hedlund LW , Nouls JC , Freeman MS , Qi Y , Driehuys B . In vivo MR imaging of pulmonary perfusion and gas exchange in rats via continuous extracorporeal infusion of hyperpolarized 129Xe. PloS One 2012;7:e31306. 22363613 10.1371/journal.pone.0031306 PMC3283644

[26] Chang YV . MOXE: a model of gas exchange for hyperpolarized 129Xe magnetic resonance of the lung. Magn Reson Med 2013;69:884–890. 22565296 10.1002/mrm.24304

[27] Stewart NJ , Leung G , Norquay G , et al. Experimental validation of the hyperpolarized 129Xe chemical shift saturation recovery technique in healthy volunteers and subjects with interstitial lung disease. Magn Reson Med 2015;74:196–207. 10.1002/mrm.25400 25106025

[28] Mansson S , Wolber J , Driehuys B , Wollmer P , Golman K . Characterization of diffusing capacity and perfusion of the rat lung in a lipopolysaccaride disease model using hyperpolarized 129Xe. Magn Reson Med 2003;50:1170–1179. 14648564 10.1002/mrm.10649

[29] Wolber J , McIntyre DJ , Rodrigues LM , Carnochan P , Griffiths JR , Leach MO , Bifone A . In vivo hyperpolarized 129Xe NMR spectroscopy in tumors. Magn Reson Med 2001;46:586–591. 11550253 10.1002/mrm.1231

[30] Nakamura K , Kondoh Y , Wakai A , Kershaw J , Wright D , Kanno I . 129Xe spectra from the heads of rats with and without ligation of the external carotid and pterygopalatine arteries. Magn Reson Med 2005;53:528–534. 15723409 10.1002/mrm.20399

[31] Miller KW , Reo NV , Schoot Uiterkamp AJ , Stengle DP , Stengle TR , Williamson KL . Xenon NMR: chemical shifts of a general anesthetic in common solvents, proteins, and membranes. Proc Natl Acad Sci U S A 1981;78:4946–4949. 6946442 10.1073/pnas.78.8.4946 PMC320304

[32] Swanson SD , Rosen MS , Coulter KP , Welsh RC , Chupp TE . Distribution and dynamics of laser‐polarized ^129^Xe magnetization in vivo. Magn Reson Med 1999;42:1137–1145. 10571936 10.1002/(sici)1522-2594(199912)42:6<1137::aid-mrm19>3.0.co;2-4

[33] Ruppert K , Brookeman JR , Hagspiel KD , Mugler JP, 3rd . Probing lung physiology with xenon polarization transfer contrast (XTC). Magn Reson Med 2000;44:349–357. 10975884 10.1002/1522-2594(200009)44:3<349::aid-mrm2>3.0.co;2-j

[34] Wolber J , Cherubini A , Leach MO , Bifone A . Hyperpolarized 129Xe NMR as a probe for blood oxygenation. Magn Reson Med 2000;43:491–496. 10748422 10.1002/(sici)1522-2594(200004)43:4<491::aid-mrm1>3.0.co;2-6

[35] Norquay G , Parnell SR , Xu X , Parra‐Robles J , Wild JM . Optimized production of hyperpolarized 129Xe at 2 bars for in vivo lung magnetic resonance imaging. J Appl Phys 2013;113:044908.

[36] Cleveland ZI , Moller HE , Hedlund LW , Driehuys B . Continuously infusing hyperpolarized 129Xe into flowing aqueous solutions using hydrophobic gas exchange membranes. J Phys Chem B 2009;113:12489–12499. 19702286 10.1021/jp9049582 PMC2747043

[37] Norquay G , Leung G , Stewart NJ , Tozer GM , Wolber J , Wild JM . Relaxation and exchange dynamics of hyperpolarized 129Xe in human blood. Magn Reson Med 2015;74:303–311. 25168398 10.1002/mrm.25417

[38] West JB . Respiratory physiology: the essentials. Philadelphia: Wolters Kluwer Health/Lippincott Williams & Wilkins; 2008 ix, 186 p.

[39] Mugler JP III , Altes TA . Hyperpolarized 129Xe MRI of the human lung. J Magn Reson Imaging 2013;37:313–331. 23355432 10.1002/jmri.23844 PMC3558952

[40] Leung G , Norquay G , Parra‐Robles J , Wild J . Measuring red blood cell oxygenation in vivo using hyperpolarized 129Xe MRI. Eur Respir J 2014;40(Suppl. 56).

[41] Kaushik SS , Freeman MS , Yoon SW , Liljeroth MG , Stiles JV , Roos JE , Michael Foster WS , Rackley CR , McAdams HP , Driehuys B . Measuring diffusion limitation with a perfusion‐limited gas—hyperpolarized 129Xe gas‐transfer spectroscopy in patients with idiopathic pulmonary fibrosis. J Appl Physiol 2014;117:577–585. 25038105 10.1152/japplphysiol.00326.2014 PMC4157168

[42] Albert MS , Kacher DF , Balamore D , Venkatesh AK , Jolesz FA . T1 of 129Xe in blood and the role of oxygenation. J Magn Reson 1999;140:264–273. 10479571 10.1006/jmre.1999.1836

[43] Savino C , Miele AE , Draghi F , Johnson KA , Sciara G , Brunori M , Vallone B . Pattern of cavities in globins: the case of human hemoglobin. Biopolymers 2009;91:1097–1107. 19365817 10.1002/bip.21201

[44] Buckingham AD , Schaefer T , Schneider WG . Solvent effects in nuclear magnetic resonance spectra. J Chem Phys 1960;32:1227–1233.

[45] Jokisaari J . NMR of noble‐gases dissolved in isotropic and anisotropic liquids. Prog Nucl Magn Reson Spectrosc 1994;26:1–26.

[46] Stephen MJ . The effect of molecular interaction on magnetic shielding constants. Mol Phys 1958;1:223–232.

[47] Jameson CJ , de Dios AC . Abinitio calculations of the intermolecular chemical shift in nuclear magnetic resonance in the gas phase and for adsorbed species. J Chem Phys 1992;97:417–434.

[48] Howard BB , Linder B , Emerson MT . Effect of dispersion interaction on nuclear magnetic resonance shifts. J Chem Phys 1962;36:485–490.

[49] Rummens FHA . Use of Bayliss‐Mcrae Dispersion Model in NMR solvent effects. Chem Phys Lett 1975;31:596–598.

[50] Zhernovaya O , Sydoruk O , Tuchin V , Douplik A . The refractive index of human hemoglobin in the visible range. Phys Med Biol 2011;56:4013–4021. 21677368 10.1088/0031-9155/56/13/017

[51] Lang G , Marshall W . Mossbauer effect in some haemoglobin compounds. J Mol Biol 1966;18:385–404. 5966294 10.1016/s0022-2836(66)80032-3

[52] Coryell CD , Pauling L , Dodson RW . The magnetic properties of intermediates in the reactions of hemoglobin. J Phys Chem 1939;43:825–839.

[53] Deninger AJ , Eberle B , Ebert M , et al. 3He‐MRI‐based measurements of intrapulmonary pO2 and its time course during apnea in healthy volunteers: first results, reproducibility, and technical limitations. NMR Biomed 2000;13:194–201. 10867696 10.1002/1099-1492(200006)13:4<194::aid-nbm643>3.0.co;2-d

[54] Cieslar K , Alsaid H , Stupar V , Gaillard S , Canet‐Soulas E , Fissoune R , Cremillieux Y . Measurement of nonlinear pO2 decay in mouse lungs using 3He‐MRI. NMR Biomed 2007;20:383–391. 17451167 10.1002/nbm.1124

[55] Deninger AJ , Eberle B , Bermuth J , Escat B , Markstaller K , Schmiedeskamp J , Schreiber WG , Surkau R , Otten E , Kauczor HU . Assessment of a single‐acquisition imaging sequence for oxygen‐sensitive 3He‐MRI. Magn Reson Med 2002;47:105–114. 11754449 10.1002/mrm.10032

[56] Kelman GR . Digital computer subroutine for the conversion of oxygen tension into saturation. J Appl Physiol 1966;21:1375–1376. 5916678 10.1152/jappl.1966.21.4.1375

[57] Severinghaus JW . Simple, accurate equations for human blood O2 dissociation computations. J Appl Physiol Respir Environ Exerc Physiol 1979;46:599–602. 35496 10.1152/jappl.1979.46.3.599

[58] Venkatesh AK , Hong KS , Kubatina L , Sun Y , Mulkern RV , Jolesz FA , Albert MS . Direct observation of the transport of 129Xe from the lung‐gas to the tissue and the blood. In Proceedings of the 9th Annual Meeting of ISMRM, Glasgow, Scotland, 2001. Abstract 0954.

[59] Ruppert K , Altes TA , Mata JF , Ruset IC , Hersman FW , Mugler JP . Detecting pulmonary capillary blood pulsations using hyperpolarized xenon‐129 chemical shift saturation recovery (CSSR) MR spectroscopy. Magn Reson Med 2015;75:1771–1780. 26017009 10.1002/mrm.25794 PMC6154503

[60] Chen RY , Fan FC , Kim S , Jan KM , Usami S , Chien S . Tissue‐blood partition coefficient for xenon: temperature and hematocrit dependence. J Appl Physiol Respir Environ Exerc Physiol 1980;49:178–183. 7400000 10.1152/jappl.1980.49.2.178

[61] Barbee JH , Cokelet GR . The Fahraeus effect. Microvasc Res 1971;3:6–16. 5092929 10.1016/0026-2862(71)90002-1

[62] Mazzanti ML , Walvick RP , Zhou X , Sun YP , Shah N , Mansour J , Gereige J , Albert MS . Distribution of hyperpolarized xenon in the brain following sensory stimulation: preliminary MRI findings. PloS One 2011;6:e21607. 21789173 10.1371/journal.pone.0021607 PMC3137603

[63] Kilian W , Seifert F , Rinneberg H . Dynamic NMR spectroscopy of hyperpolarized 129Xe in human brain analyzed by an uptake model. Magn Reson Med 2004;51:843–847. 15065259 10.1002/mrm.10726

[64] Qing K , Ruppert K , Jiang Y , et al. Regional mapping of gas uptake by blood and tissue in the human lung using hyperpolarized xenon‐129 MRI. J Magn Reson Imaging 2014;39:346–359. 23681559 10.1002/jmri.24181 PMC3758375

[65] Kaushik SS , Robertson SH , Freeman MS , He M , Kelly KT , Roos JE , Rackley CR , Foster WM , McAdams HP , Driehuys B . Single‐breath clinical imaging of hyperpolarized 129xe in the airspaces, barrier, and red blood cells using an interleaved 3D radial 1‐point Dixon acquisition. Magn Reson Med 2016;75:1434–1443. 25980630 10.1002/mrm.25675 PMC4651856

